# The impact of changes in dietary knowledge on adult overweight and obesity in China

**DOI:** 10.1371/journal.pone.0179551

**Published:** 2017-06-23

**Authors:** Lin Zhou, Qiyan Zeng, Shaosheng Jin, Guangyan Cheng

**Affiliations:** 1 Institute of Food and Nutrition Development, Ministry of Agriculture, Beijing, China; 2 School of Agricultural Economics and Rural Development, Renmin University of China, Beijing, China; 3 China Academy for Rural Development (CARD), School of Public Affairs, Zhejiang University, Hangzhou, China; Old Dominion University, UNITED STATES

## Abstract

Overweight and obesity are rapidly growing threats in China. Improvement in dietary knowledge can potentially prevent overweight and obesity, conditions which are receiving substantial attention from international organizations and governments. The purpose of this study was to investigate the impact of changes in dietary knowledge on adult overweight and obesity, using a balanced panel data consisting of 10,401 samples from the 2006, 2009, and 2011 iterations of the China Health and Nutrition Survey. Results indicate that overweight and obesity are becoming increasingly problematic in China, and the level of dietary knowledge among Chinese adults needs improvement. Moreover, the empirical results indicate that changes in dietary knowledge among adults has no significant influence on adult overweight and obesity, a likely result of lacking systematic dietary knowledge and having inadequate guidance on overweight/obesity-related behaviors.

## Introduction

With economic growth and lifestyle changes, issues of inadequate food intake in developing countries have been ameliorated; however, in turn, excess food intake has gradually become a problem in these countries [1–2]. As the prevalence of overweight and obesity has significantly increased in China, these issues have begun to catch the attention of the public and academia [3–4]. Results from the *Chinese Residents Nutrition and Chronic Disease Status Report (2015*) (From the official website of National Health and Family Planning Commission of People's Republic of China: http://www.nhfpc.gov.cn/jkj/s5879/201506/4505528e65f3460fb88685081ff158a2.shtml) reveal that the overweight rate of adults in China aged 18 and older is 30.15%, and the obesity rate is 11.9%; these rates represent increases of 7.3% and 4.8%, respectively, since 2002. Overweight and obesity are not isolated medical conditions, being linked to increased risks of a variety of chronic diseases, such as cardiovascular disease, hypertension, and Type II diabetes [5–7]. Accordingly, it is believed that the rising prevalence of overweight and obesity will put a heavy burden on the social security system. China currently spends 24.35 billion Yuan annually to address overweight, obesity, and their complications, accounting for 2.46% of China’s annual national health care expenditures [4]. Also, overweight and obesity may decrease human capital, causing huge indirect losses due to productivity differentials between obese and non-obese individuals.

However, overweight and obesity can be prevented if effective actions are taken to achieve a balance in energy intake-and-expenditure [8]. Improving dietary knowledge can help people adjust their eating and exercise behaviors to attain balance [9–12]; thus, dietary knowledge has become an important focal point in obesity prevention policies among international organizations and governments. In Japan, “The Basic Law on Food Education” has strengthened the “Food Education” program. Also, the Japanese government has established a Basic Program for Food Education Promotion to promote and implement food education in primary and secondary schools, while achieving the goal of making individuals’ diets healthier. In 2011, the United States launched *MYPlate*, a reminder-based program for healthy eating. In addition, more than 100 countries, including Australia, South Korea, and India, have developed food-based dietary guidelines that are adapted to nutritional circumstances, food availability, culinary cultures, and eating and exercise habits.

In recent decades, China has also carried out positive measures to promote dietary knowledge, aimed at guiding eating and exercise behaviors, in an effort to combat growing rates of overweight and obesity. In 2014, the *National Food and Nutrition Development Outline (2014–2020)* (From the official website of General Office of the State Council of the People's Republic of China: http://www.gov.cn/xxgk/pub/govpublic/mrlm/201402/t20140208_66624.html) was issued by the general office of the State Council. Following the guidance of the *Outline*, the first “food and nutrition week” was established in 2015, while 1-week dietary knowledge educational activities took place nationally. Additionally, some elementary and secondary schools in large cities have attempted to develop their own food and nutrition education classes. The experience and outcomes at these schools may help promote the extension of food and nutrition education classes in China.

Previous studies have explored the relationship between body mass index (BMI) and nutrition knowledge, showing mixed results. Some research done among women [13], men [14], children [15], adolescents [16], and adults [17] has shown a lack of association between BMI and nutrition knowledge, while other studies have reported a positive relationship between BMI and nutrition knowledge [18–19]. However, few studies, if any, have investigated whether dietary knowledge is associated with BMI in adults. Although both dietary knowledge and nutrition knowledge emphasize the importance of healthy eating, the former emphasizes the importance of balancing diet and exercise, while the latter generally focuses on detailed nutrient intake and diet-disease linkages. Also, we contribute to the literature by analyzing the impact of changes in dietary knowledge on overweight and obesity in China by using panel data. Although it takes time to see the impact of dietary knowledge on overweight and obesity, existing related studies build on cross-sectional data, such that trends over time have rarely been analyzed.

Therefore, the main goal of the present study was to develop an understanding of the impact of changes in dietary knowledge on adult overweight and obesity in China.

## Methods and materials

### Data

The data for this study are drawn from the China Health and Nutrition Survey (CHNS), which is compiled by the National Institute of Nutrition and Food Safety at the Chinese Center for Disease Control and Prevention and by the Carolina Population Center at the University of North Carolina. All the survey data are available on the project’s homepage (http://www.cpc.unc.edu/projects/china). The study is a longitudinal, household-based survey that began in 1989, and has been carried out in the following nine waves: 1989, 1991, 1993, 1997, 2000, 2004, 2006, 2009, and 2011. In the first eight surveys, the CHNS included eight or nine diverse provinces based on geographic position, degree of economic development, level of public resources, and residents’ health indices, which has covered four regions in China including the eastern, central, western, and northeast regions. Furthermore, an additional three “megacities” (i.e., Beijing, Shanghai and Chongqing) were added in the latest 2011 wave. A multistage random cluster process is used to draw the samples surveyed in each of the provinces. Counties in each province were stratified by income (low, middle, and high), and a weighted sampling scheme was utilized to randomly select four counties in each province. Additionally, the provincial capital and a lower income city were selected, when feasible. In two provinces, other large cities, rather than capitals, had to be selected. Four villages and townships within the counties and urban/suburban neighborhoods within the cities were also randomly selected Twenty households were selected at random in each elected community. For more information, please visit http://www.cpc.unc.edu/projects/china/about/proj_desc/survey.

The dietary knowledge survey began in 2004, and corresponding questions were appropriately adjusted in 2006. Thus, data from 2004 was ultimately eliminated to avoid error. As a result, we selected a three-wave balanced panel sample tracked from 2006 to 2011 for the panel study. Given that individuals under the age of 18 (n = 945) are undergoing rapid physical development, and given that pregnant women can gain excessive pregnancy-related weight (n = 44), our analysis includes only individuals aged 18 and over and non-pregnant individuals. Moreover, individuals suffering from a chronic disease (n = 2502) may receive professional advice from doctors, which could contribute to their dietary knowledge, and the current judgments on dietary questions in the questionnaire are not suitable for individuals suffering from chronic disease. Therefore, individuals with a chronic disease were also excluded. The final sample consisted of 10,401 adults (see S1 Dataset for detail).

### Methods

According to previous studies, the occurrence and development of overweight and obesity is the result of multiple factors including demographic, socio-economic and lifestyle variables [20–22].

Therefore, this paper establishes the model for the influence of dietary knowledge changes on adult overweight and obesity as follows:

$$Y_{ij}=\beta_{0}+\beta_{1}DK_{ij}+\beta_{2}X_{ij}+\lambda_{i}+\lambda_{j}+\mu_{ij}$$ (1)

where dependent variable *Y*_*ij*_ indicates body mass index of individual *i* in year j. *DK*_*ij*_ is the key variable, representing the level of dietary knowledge, and *β*_1_ shows the impact of *DK*_*ij*_ on *Y*_*ij*_. *X*_*ij*_ is the controlled variable; *λ*_*i*_ and *λ*_*j*_ are unobservable individual- and time-specific factors that may be correlated with *DK*_*ij*_ and *X*_*ij*_, *β*_0_ is the intercept term, and *μ*_*ij*_ is the error term.

Using CHNS panel data from 2006 to 2011, this paper used a fixed-effects model as the main method to further analyze the impact of dietary knowledge changes on adult overweight and obesity, according to the Hausman specification test.

#### Definition of adult overweight and obesity

Currently, BMI, waist circumference and waist-to-hip ratio are widely employed for classifying obesity and the risk of abdominal fat accumulation. BMI, defined as the body weight in kilograms divided by the squared body height in meters, is the most widely used indicator of adult nutritional status and the only one for which comparable data are usually available [23]; thus, BMI is used here as the indicator of adult overweight and obesity. Moreover, the following cut-offs, established by the Working Group On Obesity In China (WGOC), are used to classify the respondents: 24 kg/m^2^≤BMI<28kg/m^2^ for overweight, and BMI≥28 kg/m^2^ for obesity.

#### Measure of dietary knowledge

For the evaluation of dietary knowledge, consisting of diet and exercise, we established a comprehensive index to measure levels of dietary knowledge based on the CHNS questionnaire. The dietary knowledge component of the CHNS questionnaire includes twelve questions (Table 1). Participants’ answers on dietary knowledge are divided into “correct” and “incorrect” answers; “1” point was given for a correct answer, and “0” points for the other answers. Corresponding correct answers for each question are shown in Table 1. The scores for the 12 questions are summed up to obtain an overall score and establish a comprehensive index of dietary knowledge, representing adult respondents’ dietary knowledge at a comprehensive level.

**Table 1:** Dietary knowledge questions in the China Health and Nutrition Survey and corresponding correct answers.

Do you strongly agree, somewhat agree, somewhat disagree or strongly disagree with this statement? *Please note that the question is not asking about your actual habits	True/False
Q1: Choosing a diet with a lot of fresh fruit and vegetables is good for one’s health	T
Q2: Eating a lot of sugar is good for one’s health	F
Q3: Eating a variety of foods is good for one’s health	T
Q4: Choosing a diet high in fat is good for one’s health	F
Q5: Choosing a diet with a lot of staple foods (rice and rice products and wheat and wheat products) is not good for one’s health	T
Q6: Consuming a lot of animal products daily (fish, poultry, egg and lean meat) is good for one’s health	F
Q7: Reducing the amount of fatty meat and animal fat in the diet is good for one’s health	T
Q8: Consuming milk and dairy products is good for one’s health	T
Q9: Consuming beans and bean products is good for one’s health	T
Q10: Physical activities are good for one’s health	T
Q11: Sweaty sports or other intense physical activities are not good for one’s health	T
Q12: The heavier one’s body is, the healthier he or she is	F

Source: the dietary knowledge questionnaire is from the official website of China Health and Nutrition Survey (http://www.cpc.unc.edu/projects/china).

#### Other variables

Demographic, socio-economic, and lifestyle variables were controlled for in the models as covariates, according to previous studies [20–22]. These variables included age (in years), education level (years of formal education completed in a regular school), marital status (single = 0, otherwise = 1), income (per capital annual household income inflated to 2011, in logarithmic), daily energy intake (the average daily total energy intake during a 3-day observation), and physical activity (the intensity of physical activity). The information was collected by a questionnaire survey on individual, household community levels. Summary statistics of variables used in the present study are presented in S1 Table.

## Results

### Descriptive statistics

#### Adult obesity change trend 2006–11

**Fig 1:**
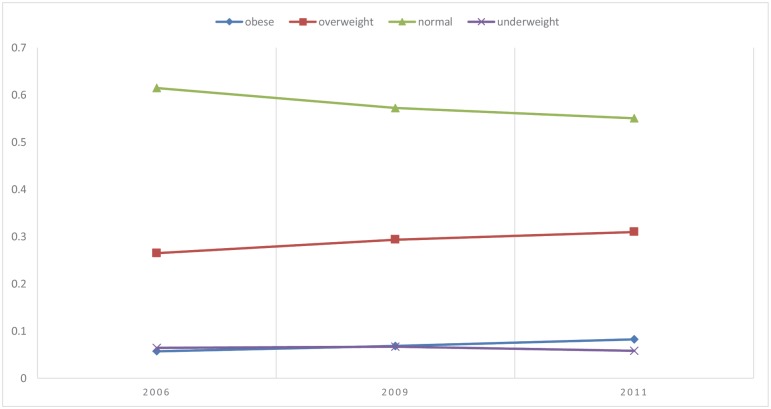
Share of population in BMI categories over time (in %).

From Table 2, we can see that mean BMI in China has increased notably, with a growth range of 0.51 from 2006 to 2011. Using the cut-off values for BMI from the Working Group On Obesity In China, Fig 1 shows that the share of overweight and obese adults has increased substantially since 2006; overweight increasing from 26.48% to 30.95% and obesity from 5.68% to 8.22%. By contrast, the share of normal-weight people has decreased from 61.44% to 55.03%. These findings demonstrate that, with the rapid development of the Chinese economy, people’s nutrition and health status have changed significantly. Accordingly, China is now struggling with a remarkable prevalence of obesity and overweight.

**Table 2:** Mean BMI and change in BMI by sex and region.

Year	Obs.	Population mean	Mean BMI by sex		Mean BMI by region	
			Male	Female	Urban	Rural
2006	3467	22.79±3.09	22.72±3.05	22.85±3.12	23.07±3.18	22.68±3.05
2009	3467	23.03±3.22	23.00±3.21	23.05±3.23	23.27±3.39	22.94±3.15
2011	3467	23.30±3.33	23.31±3.28	23.28±3.36	23.53±3.47	23.21±3.27
Change 2006–2011		+0.51	+0.59	+0.43	+0.46	+0.53

Source: Own computation based on data from the CHNS 2006, 2009 and 2011. Both mean and SD are presented for BMI.

**Fig 2:**
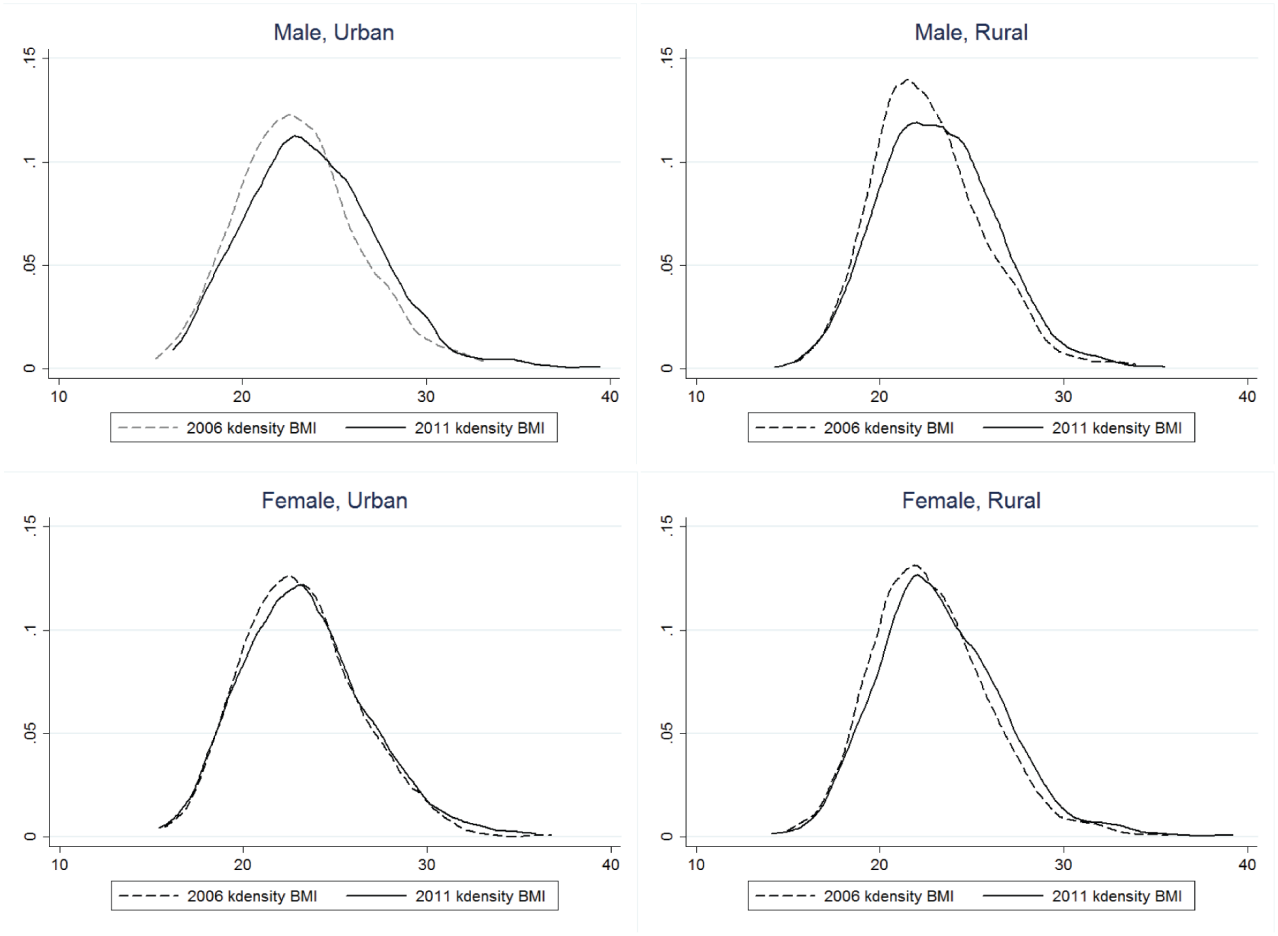
BMI density curves in 2006 and 2011.

To further analyze the differences between urban and rural areas, we can see from Table 3 that, since 2006, the mean BMI in urban areas has always been significantly higher than the mean BMI in rural areas (P<0.01); however, the gap between the two has narrowed. This shows that the issue of obesity has gradually extended from urban areas into the countryside. Fig 2 visualizes these changes over time. The urban and rural BMI probability distribution curve in 2011 is flatter than that in 2006, indicating the rising tendency of BMI; and it is worth noting that the varying amplitude of the rural curve is significantly larger than that of the urban curve, demonstrating nutrition transition, and that the growth in overweight and obesity in rural areas in recent years has been more dramatic than in urban areas. In sum, the issue of obesity in China has not been limited to urban areas, with rural areas starting to present a significant rising tendency. This patterns represents a major threat to rural areas that possess a relatively weak economic and social security base, and is a great challenge to China as a whole.

**Table 3:** Mean dietary knowledge and change in dietary knowledge by sex and region (CHNS 2006, 2009 and 2011).

Year	Obs.	Population mean	By sex		By region	
			Male	Female	Urban	Rural
2006	3467	8.68±2.37	8.77±2.25	8.61±2.47	9.11±2.33	8.52±2.37
2009	3467	8.77±2.48	8.89±2.40	8.67±2.54	9.17±2.24	8.62±2.55
2011	3467	8.83±2.44	8.79±2.44	8.86±2.43	9.36±2.16	8.63±2.50
Change 2006–2011		+0.15	+0.02	+0.25	+0.25	+0.11

Source: Own computation based on data from the CHNS 2006, 2009 and 2011. Both mean and SD values are presented for dietary knowledge.

In addition, a sex difference is noted, though it is not statistically significant (P>0.05). There has been a sustained increase in female and male mean BMI, and the mean BMI of males has been higher than that of females. Considered with Fig 2, we can also see that the change in male BMI is sharper than that of females.

#### Dietary knowledge change trend 2006–2011

From 2006 to 2011, the level of dietary knowledge among Chinese adults has improved from 8.68 to 8.83 (out of 12 questions), as shown in Table 4. Dietary knowledge levels were significantly higher among urban residents than among rural residents (P<0.01), and this trend continued over time. By contrast, in 2011, the dietary knowledge level of females surpassed that of males. Specifically, according to the distribution of level of dietary knowledge from 2006 to 2011, about 25% of adults are not able to correctly answer more than seven questions (out of 12), which demonstrates that levels of dietary knowledge among Chinese adults need improvement.

**Table 4:** Panel regressions explaining BMI (CHNS 2006, 2009 and 2011).

Independent variable	Model 1		Model 2		Model 3	
	Coefficient	SE	Coefficient	SE	Coefficient	SE
Dietary knowledge	0.006	(0.007)	-0.001	(0.006)	0.001	(0.002)
Age	0.101	(0.007) ***	0.101	(0.007) ***	0.014	(0.001) ***
Marriage	1.182	(0.201) ***	1.187	(0.201) ***	0.218	(0.042) ***
Education	-0.008	(0.010)	-0.007	(0.010)	-0.001	(0.002)
income	-0.049	(0.020) **	-0.049	(0.020) **	-0.004	(0.004)
Dairy energy intake	-2.11e-06	(0.000)	-2.15e-06	(0.000)	2.04e-06	(5.22e-06)
Light physical activity	0.080	(0.048) *	0.081	(0.048) *	0.016	(0.010)
Sample size	10,401		10,401		10,401	

Note: SE refers to the standard error, “***”, “**” and “*” means statistically significant at the 1%, 5% and 10% level.

### Regression results

Table 4 shows the regression results. As shown in model 1, changes in dietary knowledge have no effect on adult BMI. To test the robustness of our findings, we conducted two types of sensitivity analysis. In model 2, another method to measure the level of dietary knowledge was adopted as a reference. We divided the dietary knowledge questionnaire responses into *correct*, *wrong*, *neutral* or *unknown*, according to the degree of dietary knowledge mastery, with a score of “1” for a correct answer, a score of “-1” for the wrong answer, and a score of “0” for “neutral” or “unknown.” In model 3, we tested the association between dietary knowledge and overweight/obese status. Here, the dependent variable was defined as whether respondents were overweight/obese (BMI≥24 kg/m^2^); that is, overweight/obese status was defined as “1,” or otherwise “0.” Comparing the results in model 1, 2 and 3, we can see dietary knowledge has no effect on either adult BMI or overweight/obese status, while showing good robustness in our reported result. Moreover, we tested whether the dietary knowledge-BMI association differs between sex and region (urban vs. rural). As shown in S2 Table, the results demonstrate that dietary knowledge has no effects on adults BMI for females, males, urban and rural residents, respectively.

Both age and marital status have positive significant effects on BMI at a 1% statistical significance level. This is consistent with previous studies. Basal metabolic rate gradually drops and physical activity will decrease with age, which may lead to energy imbalance and increase the risks of overweight and obesity [24]. Increasingly, common sedentary lifestyles and reduced motivation to keep in shape to attract a partner after marriage mean that married men and women carry a higher risk of overweight and obesity than those who are not married [25]. Income has a negative impact on body weight changes through food purchasing power and food consumption choice [26–27]. Light physical activity is positively related to BMI (i.e., lighter physical activity levels contribute to a higher BMI). The decrease in physical activity levels may decrease energy consumption as well as the body’s demand for energy and maintaining a positive balance of the body's energy metabolism, thus increasing the risks of overweight and obesity [2,28].

## Discussion

This study’s results show that changes in dietary knowledge do not have a statistically significant effect on adult overweight and obesity in China. One of the possible reasons is that the dietary knowledge of Chinese is unsystematic and thus lacks practical guidelines. Even individuals with a relatively higher level of “fragmented dietary knowledge” cannot effectively combine dietary information with their eating and exercise behavior, let alone significantly improve their BMI. For example, it is worth noting vis-à-vis the accuracy of the 12 dietary knowledge questions that most respondents understood that eating too much high-fat food is harmful to one’s health (the average accuracy of the three surveys for this question was 76.39%), but the proportion of respondents who knew that consuming large quantities of animal products daily is not good for health has remarkably decreased (the average accuracy of the three surveys for this question was 52.99%). This outcome vividly demonstrates that dietary knowledge of Chinese adults remains unsystematic and impractical. Dietary nutrition outreach and education only emphasizes reduced intakes of high-fat foods but fails to provide specific descriptions of high-fat food categories, which results in the public being unable to combine dietary information with specific food selection. Furthermore, dietary education is still not currently part of the curriculum for Chinese students, and there are few scientific and rational channels from which to obtain accurate and systematic dietary knowledge. Therefore, individuals obtain dietary knowledge mainly from TV, internet, newspapers, and magazines; and thus the sources of information may be diffuse, short-term, or inaccurate. “Fragmented knowledge” obviously could not provide systematic guidance to eating and exercise behavior, and certainly dietary knowledge plays a limited role in preventing overweight and obesity.

Second, obesity-related information is limited or even neglected in dietary knowledge outreach. Thus, people with a relatively higher level of dietary knowledge do not necessarily have a better understanding of obesity prevention and control. For example, the *Dietary Guidelines for Chinese Residents* and the *Balanced Diet Pagoda*, as a representative of Chinese dietary knowledge outreach, have played an important role in healthy diet and exercise guidance for the general population, but the guidelines still lack obesity-related advice, and it is obvious that there is some difference between overweight or obesity individuals and normal weight individuals in patterns of diet, exercise, etc.

The lack of association between adult BMI and dietary knowledge in the present study corresponds with findings in previous studies [13–17]. With the relatively low level of dietary knowledge revealed, there appeared to be no significant differences in dietary knowledge between obese and non-obese individuals. Moreover, levels of dietary knowledge have yet to be improved when international organizations and governments treat it as an important measure for obesity prevention. We contribute to the literature by analyzing the impact of changes in dietary knowledge on overweight and obesity in China by using panel data. Although it takes time to see the impact of dietary knowledge on overweight and obesity, existing related studies build on cross-sectional data, such that trends over time have rarely been analyzed.

The present study has some limitations. First, the present study only takes into account general dietary knowledge, rather than the whole spectrum of dietary knowledge. Thus, there might be a possibility that there are some biases in accurately and objectively reflecting the level of respondents’ actual dietary knowledge. Second, although in the fixed effects models we control for unobserved heterogeneity, issues of endogeneity in the regression analysis cannot be ruled out completely. There may be potential problems of reverse causation, while suitable variables that could be used as instruments for dietary knowledge are not available in the CHNS. These limitations could be improved in future research with better data and measures on dietary knowledge.

## Conclusions

This paper has analyzed the impact of changes in dietary knowledge on overweight and obesity, using a balanced panel data of 10,401 samples from the 2006, 2009, and 2011 iterations of the CHNS. Results confirm that overweight and obesity are becoming increasingly serious matters in China. In the time period assessed, the share of the overweight has increased from 26.48% to 30.95%, and obesity has increased from 5.68% to 8.22%, while the share of normal-weight people has decreased from 61.44% to 55.03%, since 2006. The rates of overweight and obesity in rural areas has also increased dramatically, and may soon match the rates in urban areas. This trend constitutes a major threat to rural areas, which often possess a relatively weak economic and social security base, and therefore special attention must be paid to prevent and control obesity in rural areas.

Our regression results show that changes in dietary knowledge among adults has no significant influence on BMI. The main reason for this may be that adults lack systematic dietary knowledge and that there has been inadequate guidance on obesity-related behaviors. The level of dietary knowledge among Chinese adults needs to be improved. In particular, systematic dietary education is urgently needed, and it will be particularly important to incorporate a dietary course into the current compulsory education system.

## Supporting information

S1 Dataset Dataset used in this study. (DTA)

S1 Table Descriptions and basic statistics of variables (CHNS 2006, 2009 and 2011). Note: Summary statistics of variables used in the present study are presented in S1 Table based on data from the CHNS 2006, 2009 and 2011. Light physical activity includes very light physical activity (e.g. working in a sitting position, e.g., office work, watch repairer, etc.) and light physical activity (e.g., working in standing position, e. g., salesperson, laboratory technician, teacher, etc.).
(DOCX)

S2 Table Panel regressions explaining BMI between region and sex (CHNS 2006, 2009 and 2011). Note: We tested whether the dietary knowledge-BMI association differs between sex and region (urban vs. rural). As shown in S2 Table, the results demonstrate that dietary knowledge has no effects on adults BMI for females, males, urban and rural residents, respectively. SE refers to the standard error, “***”, “**” and “*” means significant at the 1%, 5% and 10% level.
(DOCX)
