# Supplementary material for: The impact of changes in dietary knowledge on adult overweight and obesity in China
Source: PLoS One. 2017 Jun 23;12(6):e0179551. doi: 10.1371/journal.pone.0179551 (PMC5482459; doi:10.1371/journal.pone.0179551)
Supplement: S1 Table — Note: Summary statistics of variables used in the present study are presented in S1 Table based on data from the CHNS 2006, 2009 and 2011. Light physical activity includes very light physical activity (e.g. working in a sitting position, e.g., office work, watch repairer, etc.) and light physical activity (e.g., working in standing position, e. g., salesperson, laboratory technician, teacher, etc.). (DOCX) [file pone.0179551.s002.docx]

**S1 Table. Descriptions and basic statistics of variables (CHNS 2006, 2009 and 2011)**

| Variables | Variable declaration | Mean | SD |
| --- | --- | --- | --- |
| BMI | The weight (kg) divided by the squared of height (m^2^) | 23.04 | 3.22 |
| Dietary knowledge | The comprehensive index to measure levels of dietary knowledge. The higher the value, the higher the level of dietary knowledge, from 0 to 12 | 8.76 | 2.43 |
| Age | In years, age over 18 | 51.34 | 13.02 |
| Marriage | 0 represents never married, 1 represents married, divorced, widowed or separated | 0.97 | 0.16 |
| Education | Years of formal education completed in a regular school | 7.15 | 4.21 |
| income | Logarithm of per capital annual household income, inflated to 2011 | 8.93 | 1.04 |
| Daily energy intake | The average daily total energy intake during three-day observation (kcal/d) | 2167.56 | 745.72 |
| Light physical activity | The intensity of physical activity, light physical activity=1, otherwise=0 | 0.45 | 0.50 |
