# Supplementary material for: The impact of changes in dietary knowledge on adult overweight and obesity in China
Source: PLoS One. 2017 Jun 23;12(6):e0179551. doi: 10.1371/journal.pone.0179551 (PMC5482459; doi:10.1371/journal.pone.0179551)
Supplement: S2 Table — Note: We tested whether the dietary knowledge-BMI association differs between sex and region (urban vs. rural). As shown in S2 Table, the results demonstrate that dietary knowledge has no effects on adults BMI for females, males, urban and rural residents, respectively. SE refers to the standard error, “***”, “**” and “*” means significant at the 1%, 5% and 10% level. (DOCX) [file pone.0179551.s003.docx]

**S2 Table. Panel regressions explaining BMI between region and sex (CHNS 2006, 2009 and 2011)**

| Independent variable | Male | | Female | | Urban | | Rural | |
| --- | --- | --- | --- | --- | --- | --- | --- | --- |
|  | Coefficient | SE | Coefficient | SE | Coefficient | SE | Coefficient | SE |
| Dietary knowledge | 0.012 | (0.011) | 0.002 | (0.010) | 0.016 | (0.016) | 0.003 | (0.008) |
| Age | 0.116 | (0.010) ^***^ | 0.089 | (0.009) ^***^ | 0.087 | (0.015) ^***^ | 0.105 | (0.008) ^***^ |
| Marriage | 1.453 | (0.229) ^***^ | 0.156 | (0.418) | 1.595 | (0.442) ^***^ | 1.051 | (0.223) ^***^ |
| Education | -0.008 | (0.014) | -0.007 | (0.014) | -0.009 | (0.022) | -0.007 | (0.011) |
| income | -0.069 | (0.030) ^**^ | -0.030 | (0.028) | -0.048 | (0.043) | -0.050 | (0.023) ^**^ |
| Dairy energy intake | -0.00004 | (0.000) | 0.0001 | (0.000) | -0.00003 | (0.000) | 3.89e-06 | (0.000) |
| Light physical activity | 0.097 | (0.074) | 0.062 | (0.063) | 0.039 | (0.104) | 0.092 | (0.054) ^*^ |
| Sample size | 4770 |  | 5631 |  | 2829 |  | 7572 |  |
